# Surface Properties of a Biocompatible Thermoplastic Polyurethane and Its Anti-Adhesive Effect against *E. coli* and *S. aureus*

**DOI:** 10.3390/jfb15010024

**Published:** 2024-01-15

**Authors:** Elisa Restivo, Emanuela Peluso, Nora Bloise, Giovanni Lo Bello, Giovanna Bruni, Marialaura Giannaccari, Roberto Raiteri, Lorenzo Fassina, Livia Visai

**Affiliations:** 1 Department of Molecular Medicine, Centre for Health Technologies (CHT), Consorzio Interuniversitario Nazionale per la Scienza e la Tecnologia dei Materiali (INSTM), Research Unit (UdR) Pavia, University of Pavia, 27100 Pavia, Italy; elisa.restivo01@universitadipavia.it (E.R.); emanuela.peluso01@universitadipavia.it (E.P.); marialaura.giannaccari01@universitadipavia.it (M.G.); livia.visai@unipv.it (L.V.); 2. Interuniversity Center for the Promotion of the 3Rs Principles in Teaching and Research (Centro 3R), University of Pavia Unit, 27100 Pavia, Italy; 3 Medicina Clinica-Specialistica, UOR5 Laboratorio di Nanotecnologie, ICS Maugeri, IRCCS, 27100 Pavia, Italy; 4 Department of Informatics, Bioengineering, Robotics and System Engineering—DIBRIS, University of Genoa, 16145 Genoa, Italy; giovanni.lobello@edu.unige.it (G.L.B.); roberto.raiteri@unige.it (R.R.); 5 Department of Chemistry, Physical Chemistry Section, University of Pavia, 27100 Pavia, Italy; giovanna.bruni@unipv.it; 6 Department of Electrical, Computer and Biomedical Engineering, Centre for Health Technologies (CHT), University of Pavia, 27100 Pavia, Italy

**Keywords:** thermoplastic polyurethane (TPU), brush, bar coater, topography, atomic force microscopy (AFM), Haralick texture analysis, bacteria, cell adhesion, hemocompatibility

## Abstract

Thermoplastic polyurethane (TPU) is a polymer used in a variety of fields, including medical applications. Here, we aimed to verify if the brush and bar coater deposition techniques did not alter TPU properties. The topography of the TPU-modified surfaces was studied via AFM demonstrating no significant differences between brush and bar coater-modified surfaces, compared to the un-modified TPU (TPU Film). The effect of the surfaces on planktonic bacteria, evaluated by MTT assay, demonstrated their anti-adhesive effect on *E. coli*, while the bar coater significantly reduced staphylococcal planktonic adhesion and both bacterial biofilms compared to other samples. Interestingly, Pearson’s R coefficient analysis showed that *R*_a_ roughness and Haralick’s correlation feature were trend predictors for planktonic bacterial cells adhesion. The surface adhesion property was evaluated against NIH-3T3 murine fibroblasts by MTT and against human fibrinogen and human platelet-rich plasma by ELISA and LDH assay, respectively. An indirect cytotoxicity experiment against NIH-3T3 confirmed the biocompatibility of the TPUs. Overall, the results indicated that the deposition techniques did not alter the antibacterial and anti-adhesive surface properties of modified TPU compared to un-modified TPU, nor its bio- and hemocompatibility, confirming the suitability of TPU brush and bar coater films in the biomedical and pharmaceutical fields.

## 1. Introduction

Microbial colonization and biofilm formation on medical devices is a major public health concern [1]. Scientists are searching for effective strategies to prevent device-associated infections because the implantable devices such as prostheses, mechanical heart valves, stents, or urinary catheters, although they may improve patients’ lives, they could be colonized by planktonic bacteria aggregating in biofilms and causing infectious diseases [2,3], which can become chronic and difficult to treat with antibiotics [4,5].

Today, the antimicrobial resistance is responsible for circa 700,000 deaths per year [6], a number that is expected to rise to 10 million by 2050, according to the World Health Organization (WHO) [7].

In the field of biomaterial science, researchers are designing devices that, thanks to their properties (e.g., surface texture), modifications (e.g., physical, chemical) [8], or presence of antimicrobial agents, could be able to hinder the bacterial adhesion [6]. Prior to the functionalization of a biomaterial with antimicrobial agents, it is very important to demonstrate its biocompatibility [9,10,11,12] and to characterize its surface properties since they can influence the cell behavior [13,14] and differentiation [15]. Properties such as topography, roughness, pore size [13], which determine the biomaterial surface texture, can affect the protein adsorption and consequently the cell adhesion [12,13,16], so that studying them is fundamental.

Various methods, such as atomic force microscopy (AFM), transmission electron microscopy (TEM), field emission scanning electron microscopy (FESEM), X-ray diffraction [17,18], and Fourier transform infrared (FTIR) spectroscopy [19], are known to study the surface characteristics. However, an interesting and innovative method, based on the analysis of an image of the material surface, is the measure of the Haralick’s features [20]: a gray-level co-occurrence matrix (GLCM) is extracted from the image and reveals the distribution of co-occurring pixel grayscale values [21]. For instance, the GLCM and its Haralick features (e.g., contrast, variance, and correlation) are used in medicine to analyze tumor heterogeneity [22], in magnetic resonance imaging (MRI) or X-ray images, as well as to predict the prokaryotic and eukaryotic cell behavior onto a biomaterial.

A very versatile biomaterial used in medical applications such as catheters, wound dressings, coatings, and drug delivery systems is represented by polyurethane (PU) [23,24,25,26], in particular by thermoplastic PU (TPU). This polymer is composed of soft and hard segments, polyols and isocyanates, respectively [24,26], whose proportions determine a different degree of flexibility, toughness, and softness [23] and confer good mechanical properties. In addition, TPUs have been shown to be durable, biocompatible, biostable [27], and hemocompatible [28,29,30], making them suitable for biomedical applications.

TPUs could be also functionalized with antibacterial molecules, including antibiotics and/or nanoparticles [31], which can be released after bacterial contact or by physicochemical surface modifications [8] to either prevent or reduce the bacterial adhesion, for example, in the medical or food industry [32], where the antimicrobial property is required [33].

In our previous works [34,35], compression-molded TPU films were prepared and characterized for different mechanical (e.g., tensile and adhesive properties) and thermal characteristics. Different agents such as titanium dioxide, chitosan and silver nanoparticles were added in the TPU mother solutions, which were used to coat the surface of TPU films, in order to provide antibacterial activities with the aim to use these materials as medical devices (e.g., probes, catheters, dynamic stents). In the cited work [35], we studied the antibacterial effect of modified polyurethane films, whose modification consisted in depositing the antibacterial coatings with brush and bar coater applicators to homogenously distribute the solution and have an equal release of antibacterial agents from the surfaces. Bare TPU solutions (non-containing antibacterial agents) were deposited with a brush and bar coater on TPU films as well to be used as control [35].

In the present work, we used the bare TPU films modified on the surface by the brush and bar coater to provide a further characterization of surface topography through AFM in order to verify whether the surface modification would not have altered the biocompatible properties of TPU Brush and Bar Coater films compared to the un-modified sample, that is, the TPU Film. We evaluated the anti-adhesive effect of the surfaces against the planktonic and biofilm cultures of Gram-negative *Escherichia coli* and the Gram-positive *Staphylococcus aureus* bacteria. Interestingly, we performed a Pearson’s R coefficient analysis, which showed that both *R*_a_ roughness and Haralick’s correlation feature were trend predictors for the adhesion of planktonic bacteria. Moreover, in this work, we provided a preliminary study of the hemocompatibility (via human fibrinogen adsorption and human platelets adhesion) and further biocompatibility characterization, not performed in the previous works, of the TPU-modified surfaces.

## 2. Materials and Methods

### 2.1. Material Preparation and Characterization

The thermoplastic polyurethane films studied in the present work were fabricated and characterized as previously described [34,35]. Briefly, TPU films were prepared by compression molding as substrate (named TPU Film) while TPU Brush and Bar Coater were obtained by depositing mother solutions of TPU using a brush and a bar coater method, respectively, which allowed a homogeneous distribution of the polymer solutions. The materials’ characterizations like NMR spectroscopy, X-ray diffraction, thermogravimetric analysis, differential scanning calorimetry as well as the wettability, have been described in our previous works. In particular, the characterization analyses of TPU samples including the main tensile properties (elastic modulus (*E* = 26.2 ± 1.4 MPa), maximum stress (*σ*_max_ = 36.4 ± 1.6 MPa), elongation at break (*ε*_break_ = 1075 ± 44%), and wettability (θ = ~90°) were published in the previous work [34], while the adhesion test results (maximum force ~−0.3 N for compression and ~0.3 N for tension) of the bare TPU mother solution were published in [35]. For further characterization, refer to the cited works.

### 2.2. Scanning Electron Microscopy (SEM)

TPU film, brush, and bar coater were observed using a Zeiss EVO-MA10 scanning electron microscope (Zeiss, Oberkochen, Germany) with an accelerating voltage of 20 kV, at 10k× and 40k× magnification.

### 2.3. Atomic Force Microscopy (AFM)

AFM topography images were obtained using a Nanowizard 4XP AFM (Bruker Nano GmbH, Berlin, Germany) coupled to an upright optical microscope (Axio Zoom.V16, Carl Zeiss, Iena, Germany). All measurements were conducted at room temperature (RT) in water solution (NaCl 0.9%). Topography was measured in contact mode, using a commercial AFM rectangular cantilever characterized by a conical tip with a hard diamond-like coating in order to prevent wearing over different scans (model HQ:CSC17/Hard/AI BS, μ-Masch, Tallinn, Estonia). The tip radius is less than 20 nm with a full cone angle of 40° and a nominal tip height of 15 μm. The cantilever spring constant was determined by means of the Sader method [36] and resulted to be K = 0.18 N/m. For each sample, topography images (512 × 512 pixel) were collected on at least ten different, randomly selected, 100 × 100 μm^2^ areas using a force setpoint of 30 nN.

AFM images were processed using the instrument software (JPKSPM Data Processing) in order to remove tilt and calculate three surface roughness parameters: *R*_a_ (Arithmetic Average Roughness), *R*_q_ (Root Mean Square Roughness), and *R*_t_ (Maximum Peak-to-Valley Roughness).

### 2.4. Bacterial Cell Adhesion and Biofilm Formation

#### 2.4.1. Bacterial Strains and Culture Conditions

The used microbial strains were *Escherichia coli* ATCC (American Type Culture Collection, Manassas, VA, USA) 25922 (*E. coli*) and *Staphylococcus aureus* ATCC 25923 (*S. aureus*). *E. coli* bacteria were grown in Luria Bertani (LB) broth (ForMedium, Norfolk, UK), overnight, under aerobic conditions at 37 °C using a shaker incubator (VDRL Stirrer 711/CT, Asal S.r.l., Milan, Italy) and *S. aureus* in Tryptic Soy Broth (TSB) (ForMedium). The number of bacterial cells/mL of both cultures was determined by comparing the optical density (OD_600_) of the sample with a standard curve relating the OD to the cell number [37].

#### 2.4.2. MTT Assay

Bacteria (10^5^/sample) were inoculated for 6 h at 37 °C on sterile TPU samples and in tissue culture plates (TCP) used as control. Planktonic bacteria contained in the supernatant, after the desired incubation time, were removed and the samples were gently washed with PBS 1×. They were transferred in clean wells where the viability of adherent bacteria was evaluated through 3-(4,5-dimethylthiazol-2-yl)-2,5-diphenyltetrazolium bromide (MTT) colorimetric assay (Sigma-Aldrich, St. Louis, MO, USA) as described in our previous work [35]. The experiment was performed in triplicate and repeated twice.

#### 2.4.3. Biofilm Formation

Overnight cultures of bacteria were diluted to 10^7^/sample in LB containing 0.5% glucose for *E. coli* and 0.25% for *S. aureus* [38] and incubated for 24 h at 37 °C on TPU film, brush, and bar coater samples contained in 96-well culture plates (Euroclone S.p.a., Pero, Italy). After the incubation time, the surfaces were washed and transferred, and the biofilm viability assay was performed as previously described. The experiment was performed in triplicate and repeated twice.

### 2.5. Texture Analysis of SEM Images

The texture of a gray-level image can be calculated through Haralick features; therefore, it is possible to correlate these data with the observed biological parameters, namely the number of bacteria in planktonic culture. For each SEM image of the materials without bacteria, we have selected at least two regions of interest (ROIs) to measure the gray-level co-occurrence matrix (GLCM) [39]. Then, for each GLCM, we have calculated one Haralick’s feature: the “correlation” [20]. The correlation computes the amount of similarity inside the GLCM and is a measure of the image’s pixel homogeneity.

### 2.6. Platelets’ Adhesion

Human platelet-rich plasma (hPRP) was obtained from Fondazione IRCCS Policlinico San Matteo, Pavia (Italy). hPRP was isolated according to “Decreto Ministero della Salute 2 November 2015 n.69, Disposizioni relative ai requisiti di qualità e sicurezza del sangue e degli emocomponenti” and “Accordo Stato-Regioni n.225/CSR 13 December 2018, Schema-tipo di convenzione per la cessione del sangue e dei suoi prodotti per uso di laboratorio e per la produzione di dispositivi medico-diagnostici in vitro”. The quantification of platelets’ adhesion to TPU samples was determined through lactate dehydrogenase (LDH) assay (Sigma-Aldrich). Human platelets were diluted in 10 mM EDTA (VWR Chemicals, Milan, Italy) at a concentration of 2 × 10^8^ platelets/mL and seeded on sterile samples (film, brush, bar coater) for 1 h at 37 °C [29]. After the incubation time, the supernatant was removed, the samples washed three times with sterile PBS 1× and transferred into clean Eppendorfs. Lysis of adherent platelets was performed with 300 µL of 1% Triton X-100 on ice [29], for 30 min. The Eppendorfs were centrifuged at 15,000 rpm, for 30 min, at 4 °C and the supernatant was used to quantify LDH release according to the manufacturer’s instructions. A titration curve with known concentration of platelets/mL was used to plot the obtained absorbance.

### 2.7. Enzyme-Linked Immunosorbent Assay (ELISA)

Human fibrinogen (10 µg/mL) was immobilized on the three TPU types, overnight at 4 °C, on agitation. The wells were washed three times with PBST (PBS 1× + 0.05% Tween 20) and then blocked with BSA (bovine serum albumin) 3% in PBST at RT. After that, anti-fibrinogen-HRP conjugated antibody (1:10,000) (Rockland Immunochemicals Inc., Pottstown, PA, USA) was incubated for 1 h at RT, on agitation. The wells were washed, and the reaction was developed through OPD tablets (Sigma-Aldrich). The absorbance was read at 450 nm with a reference wavelength of 620 nm [12]. The obtained absorbance was related to a calibration curve containing known amounts of fibrinogen and expressed as [µg/mL]/cm^2^.

### 2.8. Fibroblasts’ Viability

NIH-3T3 murine fibroblast cell line (ATCC CRL-1658) was obtained from the American Type Culture Collection (ATCC, Manassas, VA, USA) and cultured as described in [40]. They were seeded either in wells or onto TPU samples to evaluate the biocompatibility of TPU materials.

#### 2.8.1. Indirect Experiment

DMEM medium was incubated overnight at 37 °C + 5% CO_2_ on TPU Film, Brush, and Bar Coater to evaluate the sample’s cytotoxicity. At the end of the incubation time, the solution was 2-fold serial diluted and incubated with NIH-3T3 cells (2 × 10^4^/well) for 24 h at 37 °C + 5% CO_2_. After incubation, the cells were washed with PBS 1× and incubated with MTT (Sigma-Aldrich) for 3 h at 37 °C + 5% CO_2_ [41]. The reaction was read at 595 nm with the reference wavelength of 655 nm. Titration curve interpolation was used to express the number of cells in each sample. The results were normalized to the number of cells grown in a tissue culture plate (TCP), which was used as a control.

#### 2.8.2. Direct Experiment

NIH-3T3 cells (6 × 10^4^) were seeded on TPU film, brush, and bar coater for 24 h at 37 °C + 5% CO_2_. Viability of cells was evaluated through MTT as previously described.

### 2.9. SEM of Cells and Platelets

Bacteria (planktonic cultures and biofilms), platelets, and fibroblasts were incubated on the three surfaces as previously described. After the desired incubation time, the samples were gently washed with PBS 1× and fixed with glutaraldehyde 2.5% (Sigma-Aldrich) and treated as described in [12]. Images of bacterial planktonic adhesion were acquired at 6k× and 15k× magnifications, with biofilms acquired at 6k× and 30k×, platelets at 3k× and 10k×, and fibroblasts at 3k×.

### 2.10. Statistical Analysis

Statistics regarding biological data was carried out by considering the mean of the results (in triplicate) obtained from two separate experiments. The analysis was performed using GraphPad Prism 9 (GraphPad Inc., Boston, MA, USA). The analysis was performed using Student’s unpaired, two-sided *t*-test (significance level of 0.05) in comparison to the TCP control. In addition, one-way analysis of variance (ANOVA), followed by Bonferroni’s multiple comparisons test, was performed [12].

## 3. Results

### 3.1. Evaluation of Morphological and Topographical Properties of TPUs

**Figure 1:**
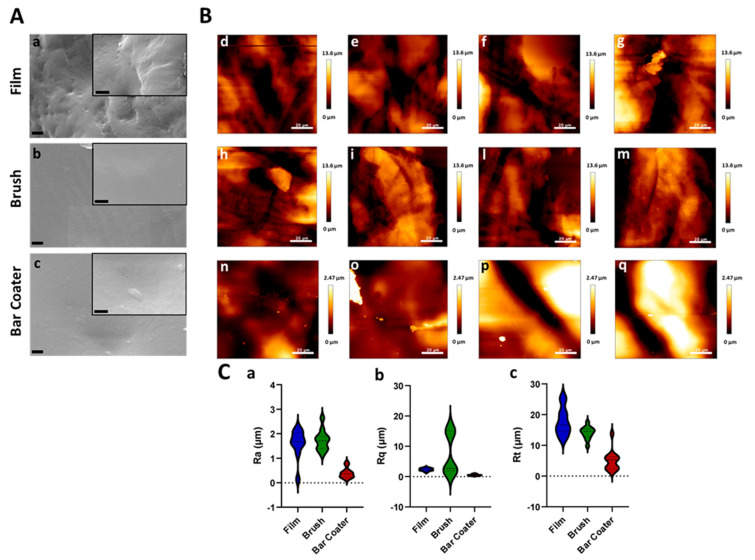
Thermoplastic polyurethane surface. Microscopic images (**A**) of thermoplastic polyurethane (TPU) Film (**a**), Brush (**b**), and Bar Coater (**c**). SEM images were acquired at 10k× magnification (scale bar 2 µm), and insets at 40k× (scale bar 1 µm). Morphological analysis (**B**) in four different areas of TPU Film (**d**–**g**), Brush (**h**,**i**,**l**,**m**), and Bar Coater (**n**,**o**,**p**,**q**). Roughness values (**C**) calculated for the three samples on *n* = 10 images (topography 100 × 100 µm^2^). Average Roughness *R*_a_ (**a**), RMS Roughness *R*_q_ (**b**), Peak-to-Valley Roughness *R*_t_ (**c**) for Film (blue), Brush (green), and Bar Coater (red).

Scanning electron microscopy (SEM) and atomic force microscopy (AFM) were used to visualize the surface (Figure 1A) and the topography (Figure 1B) of the TPU Film (a,d–g), Brush (b,h–m), and Bar Coater (c,n–q). Figure 1B reports representative images of the topography of each of the three samples under investigation: Film (d–g), Brush (h–m), and Bar Coater (n–q). Images were obtained by scanning a 100 × 100 μm^2^ area in different regions over the surface. No sample-distinctive structural features could be observed. Yet it can be observed that the bar coater-modified surface (n–q) looked flatter than the others. The roughness of the surface was calculated through AFM (C).

Figure 1C shows the distributions of the three surface roughness parameters that have been measured from each topography image; it can be observed that the lowest roughness values were obtained for the TPU Bar Coater, providing an indication of a smoother surface among the three samples.

### 3.2. Evaluation of Planktonic Bacterial Adhesion on TPUs

**Figure 2:**
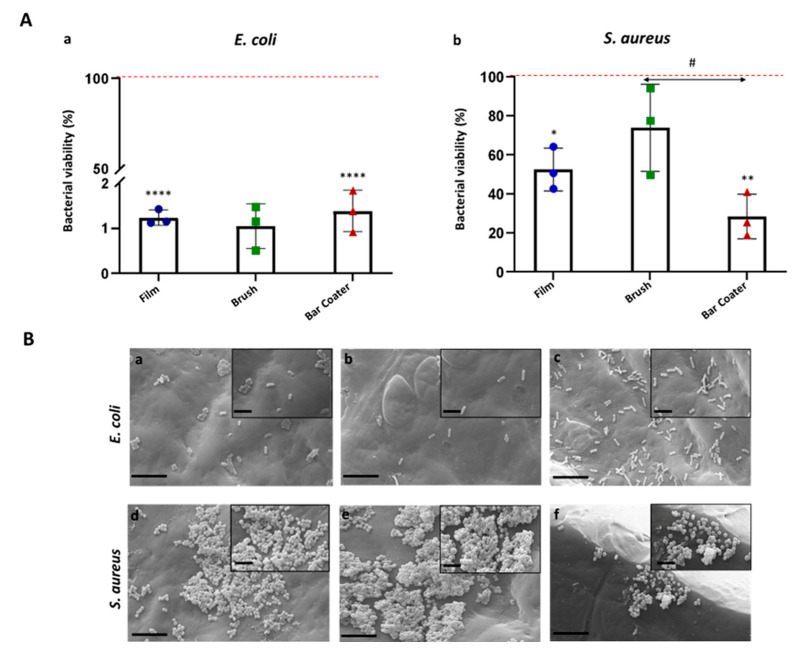
Bacterial adhesion on TPUs. *E. coli* (**A**, **a**; **B**, **a**–**c**) and *S. aureus* (**A**, **b**; **B**, **d**–**f**) were incubated for 6 h at 37 °C on Film, Brush, and Bar Coater. After removal of supernatant, the viability of adherent bacteria has been determined through MTT (**A**). Cell viability (%) was represented with respect to the TCP control, consisting of bacteria grown in medium and set as 100% (red line). Data are represented as the mean values of the replicates (*n* = 3) ± the standard deviation (SD), represented by the error bars. Statistical analysis (*, #) indicates the analysis vs. TCP: *p* < 0.0001 (****), *p* < 0.01 (**), *p* < 0.05 (*). One-way analysis of variance (ANOVA), followed by Bonferroni’s test between samples (#) within bacterium (*p* < 0.05), was performed. Data not significant (*p* > 0.05) for *E. coli*. SEM images (**B**) of *E. coli* (**a**–**c**) and *S. aureus* (**d**–**f**) bacterial adhesion on Film (**a**,**d**), Brush (**b**,**e**) and Bar Coater (**c**,**f**) were acquired at 6k× magnification (scale bar 8 µm), and insets at 15k× (scale bar 2 µm).

The ability of planktonic bacteria to adhere on the TPU Film, Brush, and Bar Coater was evaluated through an MTT viability assay (Figure 2A). Figure 2 shows the *E. coli* viability (A,a) and distribution (B,a–c) on the three samples after 6 h of adhesion time, whereas in panels (A,b) and (B,d–f) *S. aureus* data are reported.

Figure 2A shows that only 1% of Gram-negative *E. coli* (a) was viable after 6 h of adhesion on TPU with respect to the TCP control, represented by bacteria which adhered on the well surface. The non-adhesive properties of TPU against *E. coli* are independent from the type of deposition method. Panel (b) shows, on the contrary, the opposite behavior of Gram-positive *S. aureus* on TPUs: *S. aureus* was viable on Film (~50%), Brush (~70%), and Bar Coater (~25%). A comparative summary table (Table 1) is reported as follow. The data are supported by SEM images (Figure 2B).

### 3.3. TEXTURE Analysis of the SEM Images for the Prediction of the Bacteria Number in Planktonic Cultures

**Figure 3:**
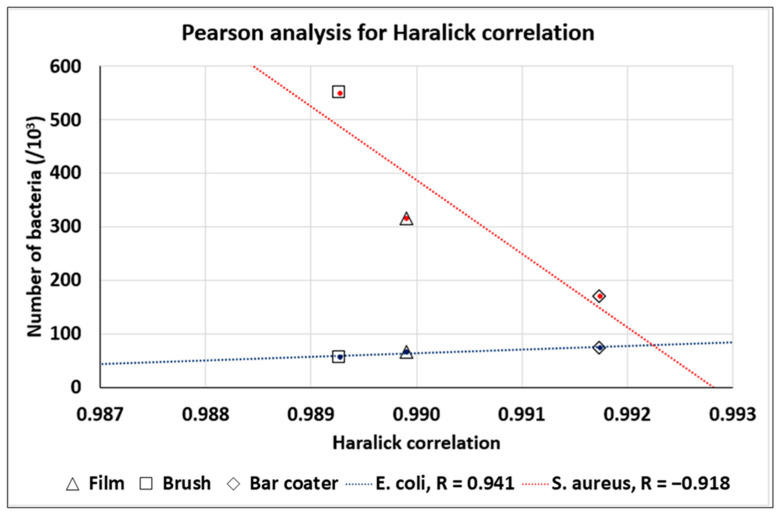
Pearson analysis for Haralick correlation.

**Figure 4:**
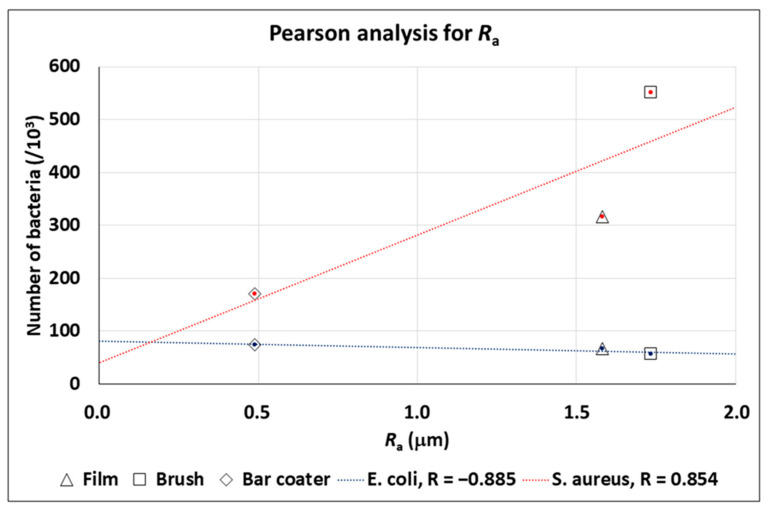
Pearson analysis for rugosity *R*_a_.

The Haralick’s texture analysis was performed on the TPU Film, Brush, and Bar Coater. SEM images, at 40k× magnification, were used to extract the Haralick correlation feature and to analyze the link between this feature and the bacterial adhesion (Figure 3). Moreover, we have correlated the *R*_a_ roughness to the bacterial adhesion (Figure 4).

In order to link the Haralick correlation feature of the material surface to the number of bacteria in planktonic culture onto that surface, we have performed a Pearson analysis: the Pearson R coefficient is the most common method to study a linear relationship; it is a number between −1 and 1 that measures the strength and the direction of the relationship between two variables, in our work, the Haralick correlation feature and the number of bacteria in planktonic culture.

In Figure 3, we can see that the Haralick correlation is a trend predictor for the number of bacteria (in fact, |R| > 0.9 for both bacteria). In particular, for *E. coli*, the number of bacteria increases with an increasing Haralick’s correlation; on the other hand, for *S. aureus*, the number of bacteria decreases with an increasing Haralick’s correlation. In addition, major differences were found for the brush-modified surface, whereas minor differences were found for the bar coater-modifiedsurface.

Figure 4 reports the rugosity *R*_a_, which is a trend predictor for the number of bacteria (in fact, |R| > 0.85 for both bacteria). In particular, for *E. coli*, the number of bacteria decreases with an increasing rugosity *R*_a_; on the other hand, for *S. aureus*, the number of bacteria increases with an increasing rugosity *R*_a_. In addition, major differences were found for the Brush surface, whereas minor differences were observed for the Bar Coater surface.

### 3.4. Effect of TPU Surfaces on Bacterial Biofilms

**Figure 5:**
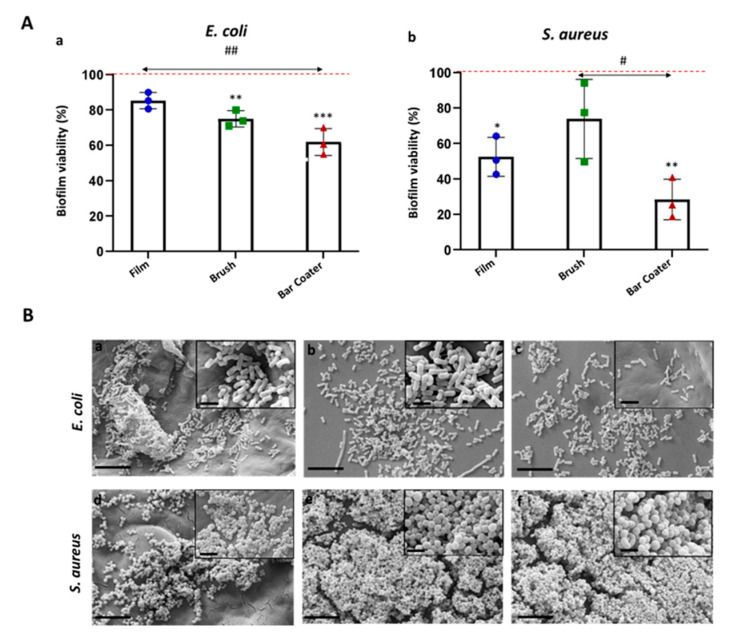
Bacterial biofilm on TPUs. *E. coli* (**A**, **a**; **B**, **a**–**c**) and *S. aureus* (**A**, **b**; **B**, **d**–**f**) were incubated in biofilm conditions on TPU Film, Brush, and Bar Coater for 24 h at 37 °C. After removal of supernatant, viability of biofilms was determined through MTT (**A**). Biofilm viabilities (%) were reported with respect to the TCP control, represented by biofilm grown in medium and set as 100% (red line). Data are represented as the mean values of the replicates (*n* = 3) ± the standard deviation (SD), represented by the error bars. Statistical analysis (*, #) indicates the analysis vs. TCP: *p* < 0.001 (***), *p* < 0.01 (**), *p* < 0.05 (*). One-way analysis of variance (ANOVA), followed by Bonferroni’s test between samples (#) within bacterium, was performed: *p* < 0.05 (#) and *p* < 0.01 (##). SEM images (**B**) of *E. coli* (**a**–**c**) and *S. aureus* (**d**–**f**) biofilms on Film (**a**,**d**), Brush (**b**,**e**), and Bar Coater (**c**,**f**) were acquired at 6k× magnification (scale bar 8 µm), and insets at 30k× (scale bar 2 µm).

After the adhesion of planktonic bacteria, we have evaluated the ability of that bacteria to form biofilms on the surfaces. Figure 5 shows that the bacterial biofilms grew on all samples, although the bar coater-modified surface is able to reduce the *E. coli* biofilm viability by circa 40% (A,a) and the *S. aureus* biofilm viability by circa 80% (A,b, Table 1). The film surface can reduce the *E. coli* biofilm by circa 20% and the staphylococcal one by circa 50%. The brush-modified surface, instead, showed the same percentage of reduction (circa 30%) for both bacterial biofilms (A, Table 1). Panel B shows the SEM images of the bacterial biofilms.

As reported in numerous papers in the literature [13,42,43], the surface roughness is often correlated with high levels of biofilm formation as it increases the surface area available for bacterial attachment. This helps to explain why, particularly on the TPU Brush samples (which had the highest roughness compared to the Film and Bar Coater samples), the biofilm viability of *S. aureus* was higher.

### 3.5. Evaluation of TPU Surface Effect on Platelets, Fibrinogen, and Cells Adhesion

To further characterize the biological properties of the TPU surfaces, the adhesion of platelets, fibrinogen [44,45], and eukaryotic cells was evaluated [46].

**Figure 6:**
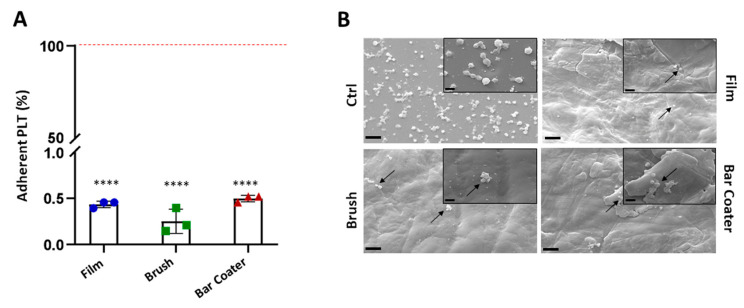
Platelets adhesion. Platelets (PLT) were incubated for 1 h at 37 °C + 5% CO_2_ on TPU samples: Film, Brush, and Bar Coater. Viability has been determined through LDH assay on supernatant (**A**). Data are represented as percentage of adherent platelets on TPUs with respect to the TCP control, denoted by the platelets seeded in a well and set as 100% (red line). Data are shown as the mean values of the replicates (*n* = 3) ± the standard deviation (SD), represented by the error bars. Statistically significant differences of samples vs. TCP were reported: *p* < 0.0001 (****). One-way analysis of variance (ANOVA), followed by Bonferroni’s test between samples, showed no significant difference between samples. After removal of supernatant, the adherent platelets on TPU surfaces and on control glass were fixed and the SEM images (**B**) were acquired at 3k× magnification (scale bar 20 µm), and insets at 10k× (scale bar 2 µm). Platelets are indicated by arrows on the TPU samples. No pseudopodia, characteristics of activated PLTs, were observed on either the control or the samples.

Figure 6 shows data regarding platelets seeded for 1 h at 37 °C on the TPU Film, Brush, and Bar Coater compared to the TCP control, represented by platelets adhered on a well (red line). The data demonstrate that there is no significant difference between samples and all three surfaces did not allow platelets to adhere (A) (<0.5% adhesion vs. TCP). The results were due to the hydrophobicity of TPU, already reported by Villani et al. [34], and our findings are in accordance with the literature [44,45]. Quantitative data are supported by SEM images (B). No activation of platelets was observed on either the control or samples, since no bulbous and pseudopodia were present on the adhered platelet’s surface [46,47,48].

**Figure 7:**
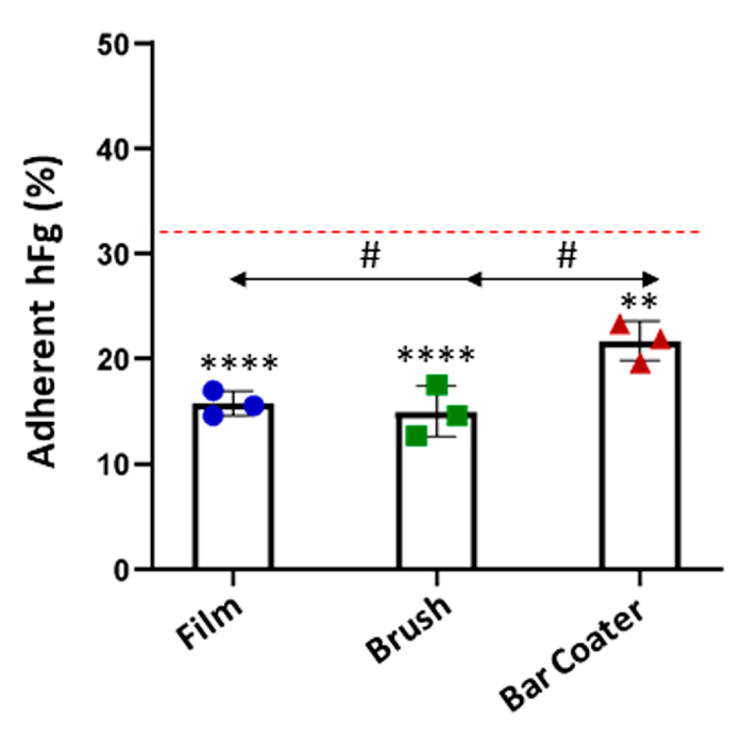
Fibrinogen quantification. Human fibrinogen (hFg) was incubated, overnight at 4 °C, on TPU Film, Brush, and Bar Coater. The adherent protein on TPU was detected through an anti-fibrinogen HRP-conjugated antibody and quantified via ELISA assay. Data are represented as percentage of adherent hFg on TPU materials with respect to the TCP control (red line). Data are shown as the mean values of the replicates (*n* = 3) ± the standard deviation (SD), represented by the error bars. Statistically significant differences of samples vs. TCP were reported: *p* < 0.0001 (****), *p* < 0.01 (**). One-way analysis of variance (ANOVA), followed by Bonferroni’s test between samples (#), was performed: *p* < 0.05.

Furthermore, the capacity of fibrinogen to adhere on modified TPU surfaces was evaluated, since the importance for platelets adhesion. The results, shown in Figure 7 and Table 1, confirmed that a low percentage, with respect to the TCP control, could bind to the surface [44]. There is no significant difference between film and brush, whose surfaces displayed circa 15% of fibrinogen adhesion. The Bar Coater surface, on the contrary, demonstrated an ability to bind circa 20% of the protein compared to TCP control.

**Figure 8:**
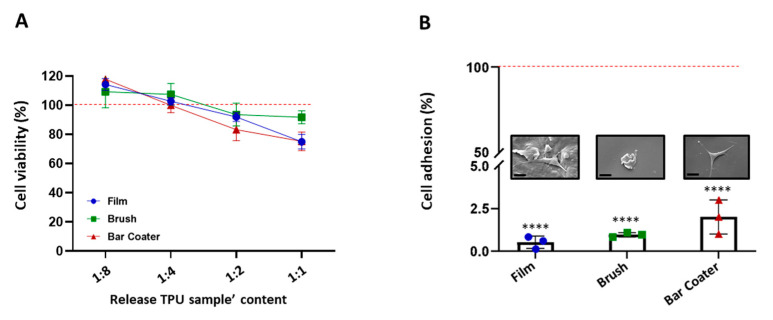
Cell viability (**A**) and adhesion (**B**). NIH-3T3 cell medium was incubated with TPU samples overnight at 37 °C + 5% CO_2_. The released content of polymer from the samples was 2-fold diluted and incubated with NIH-3T3 cells for 24 h. The viability was evaluated through MTT colorimetric test (**A**). Data are shown as percentage of cell viability with respect to TCP control (red line) represented by cells grown in a well. Statistical analysis reported no significant differences (*p* > 0.05) with respect to TCP control and between samples. Cells were seeded on TPU samples for 24 h at 37 °C + 5% CO_2_ (**B**). The viability of adherent cells was evaluated through MTT. Data are shown as percentage of cell adhesion with respect to TCP control (red line). Data are represented as the mean values of the replicates (*n* = 3) ± the standard deviation (SD), denoted by the error bars. Statistics indicates the analysis vs. TCP: *p* < 0.0001 (****). ANOVA analysis was performed, and no significant differences (*p* > 0.05) were observed. SEM images of the cell adhesion on TPU surfaces were acquired at 3k× (scale bar 20 µm).

Finally, the biocompatibility and the adhesive properties of TPU vs. NIH-3T3 fibroblasts were assessed [49,50] and are shown in Figure 8 and summarized in Table 1. Panel A reports data obtained from the indirect cytotoxicity assay, where the content of TPU, released overnight in the cell medium, was tested on cells. As shown in the figure, only the undiluted solutions, recovered from Film and Bar Coater, reduced the fibroblast viability of circa 20% with respect to the TCP control (red line), represented by cells grown in a well. On the other hand, the more diluted solutions were not toxic for the cells, as illustrated in the figure. No significant differences between samples were observed. Finally, panel B reports the percentage and the SEM images of NIH-3T3 cells’ adhesion onto the three surfaces. Fibroblasts’ adhesion on all surfaces was <2.5% with respect to TCP control. Significant differences were observed with respect to the TCP. The very low fibroblast adhesion was due to the hydrophobic nature of the TPU [35], which led to a reduction in protein adsorption (Figure 7) and, consequently, to low cell adhesion [51].

## 4. Discussion

The development of biomaterials with specific characteristics is crucial for their application in medicine and in tissue engineering. Surface topography as well as wettability [13] play an important role in prokaryotic and eukaryotic cells’ adhesion.

In this work, we studied whether the different deposition methods of a TPU solution, distributed by brush and bar coater applicators on TPU substrates (TPU film) [35], would have altered the TPU-modified surfaces’ topography and would have affected their anti-adhesive and biological properties.

In this study, the texture analysis of three surfaces has been performed by AFM, which provided information regarding the TPU-modified surfaces’ roughness, compared to the un-modified one (TPU Film). The roughness parameters [13,52], which measure the different height between areas of a surface [53], are important for evaluating prokaryotic and eukaryotic cells-biomaterial interaction.

The obtained AFM topography data confirmed that no characteristic traits were present on the surfaces. They were smooth, but the bar coater-modified surface was flatter than the others, probably due to the type of TPU deposition [35]. What further bolstered our findings was the fact that the bar coater sample displayed a notably narrower dispersion of results when compared to Brush and Film. As reported by the literature [2,54] the different type of deposition methods can vary the roughness of the surface and, consequently, the interaction with cells. The anti-adhesive effect of the modified surfaces was evaluated against planktonic cultures of Gram-negative *E. coli* and Gram-positive *S. aureus*. Briefly, the viability of planktonic adherent bacteria was assessed after 6 h vs. the TCP control, represented by bacteria grown in a well. The obtained data demonstrated the anti-adhesive effect of Film, Brush and Bar Coater surfaces against *E. coli*. Staphylococcal cells, instead, displayed major adhesion on the brush-modified surface and minor adhesion on the Bar Coater’s. As known in the literature, the surface characteristics such as roughness, wettability (hydrophilicity and hydrophobicity), play a fundamental role for cell adhesion [13,52]. A better adhesion of prokaryotic and eukaryotic cells, indeed, is observable both on rough surfaces, since they present more areas to let cells anchor [55], and on hydrophilic surfaces [13]. For this reason, we explain why the higher staphylococcal adhesion is observable on the brush-modified surface [2]. However, the opposite bacterial behavior, displayed by planktonic *E. coli* and *S. aureus* bacteria, could be explained by their different characteristics mainly in the cell wall and motility [56,57,58] since they belong to Gram-negative and Gram-positive strain, respectively. Interestingly, we performed Pearson’s R coefficient analysis, which showed that both *R*_a_ roughness and Haralick correlation feature [59,60,61], were trend predictors for the adhesion of planktonic bacterial cells.

We evaluated the ability of adherent bacteria to form biofilms, which are complex microbial communities protected by a self-produced polysaccharide matrix [2,62,63]. The obtained data showed that the brush-modified surface favored, as supported by the literature [2], the formation of both biofilms, whereas the bar coater’s smooth surface reduced them. The surfaces, since they did not contain antibacterial agents, were not able to inhibit the formation of both biofilms, as reported in the literature [64].

Furthermore, we evaluated the platelets’ adhesion and the fibrinogen adsorption on brush- and bar coater-modified surfaces, and their biocompatibility for a potential use as coatings in cardiovascular devices. Our findings showed that both surfaces, compared to the TPU Film, did not release any toxic compound for cells [28]. Moreover, from platelets’ adhesion and fibrinogen adsorption analyses [27,28,29], important for a preliminary evaluation of the hemocompatibility [65], and from the assessment of fibroblasts’ adhesion, we confirmed the anti-adhesive effect of TPU and of its brush- and bar coater-modified surfaces. These results are supported by the hydrophobic nature of the TPU [34], which causes proteins to be adsorbed onto the surface in a denatured state [13], not allowing platelets [27,28,29] and fibroblasts to adhere [66].

## 5. Conclusions

In this study, a brush and bar coater, both interesting for the deposition of polymer solutions on TPU films, were able to modify the surface topography of the TPU material by changing the surface roughness. However, these changes did not significantly alter the anti-adhesive properties of the TPU-modified surfaces, as well as their ability to hinder human fibrinogen adsorption, human platelets’, and fibroblasts’ adhesion. However, further analyses, both in vitro and in vivo, will be required to confirm all this experimental evidence. Finally, using Pearson’s analysis to correlate the bacterial adhesion with roughness data and with Haralick’s correlation feature, we confirmed how important it is to have a good characterization of the surface as a predictor of the cell–material interaction.

## Figures and Tables

**Table 1:** Summary of the adhesion onto TPU surfaces.

TPU Surface	Adhesion						
	Planktonic Behavior Predicted by Haralick Analysis		Biofilm		PLTs	hFg	NIH-3T3
	*E. coli*	*S. aureus*	*E. coli*	*S. aureus*			
Film	~1%	~50%	80%	50%	<0.5%	~15%	<2.5%
Brush	~1%	~70%	70%	70%	<0.5%	~15%	<2.5%
Bar Coater	~1%	~25%	60%	20%	<0.5%	~20%	<2.5%

## Data Availability

The data presented in this study are available on request from the corresponding author.
